# Global warming may significantly increase childhood anemia burden in sub-Saharan Africa

**DOI:** 10.1016/j.oneear.2023.09.003

**Published:** 2023-10-20

**Authors:** Yixiang Zhu, Cheng He, Antonio Gasparrini, Ana Maria Vicedo-Cabrera, Cong Liu, Jovine Bachwenkizi, Lu Zhou, Yuexin Cheng, Lena Kan, Renjie Chen, Haidong Kan

**Affiliations:** 1 School of Public Health, Key Lab of Public Health Safety of the Ministry of Education and NHC Key Lab of Health Technology Assessment, Fudan University, Shanghai 200032, China; 2 IRDR ICoE on Risk Interconnectivity and Governance on Weather/Climate Extremes Impact and Public Health, Fudan University, Shanghai 200438, China; 3 Helmholtz Zentrum Mu€nchen - German Research Center for Environmental Health (GmbH), Institute of Epidemiology, Neuherberg, Germany; 4 Department of Public Health, Environments and Society, London School of Hygiene & Tropical Medicine, London, UK; 5 Centre for Statistical Methodology, London School of Hygiene and Tropical Medicine, London, UK; 6 Centre on Climate Change and Planetary Health, London School of Hygiene and Tropical Medicine, London, UK; 7 Institute of Social and Preventive Medicine, University of Bern, Bern, Switzerland; 8 Oeschger Centre for Climate Change Research, University of Bern, Bern, Switzerland; 9 Department of Environmental and Occupational Health, Muhimbili University of Health and Allied Sciences, Dar es Salaam, Tanzania; 10 Department of Hematology, The First People’s Hospital of Yancheng, Yancheng Affiliated Hospital of Xuzhou Medical University, The Fourth Affiliated Hospital of Nantong University, Yancheng, China; 11 Bloomberg School of Public Health, Johns Hopkins University, Baltimore, MD, USA; 12 Children’s Hospital of Fudan University, National Center for Children’s Health, Shanghai, China

## Abstract

Childhood anemia constitutes a global public health problem, especially in low- and middle-income countries (LMICs). However, it remains unknown whether global warming has an impact on childhood anemia. Here, we examined the association between annual temperatures and childhood anemia prevalence in sub-Saharan Africa and then projected childhood anemia burden attributable to climate change. Each 1°C increment in annual temperature was associated with increased odds of childhood anemia (odd ratio = 1.138, 95% confidence interval: 1.134–1.142). Compared with the baseline period (1985–2014), the attributable childhood anemia cases would increase by 7,597 per 100,000 person-years under a high-emission scenario in the 2090s, which would be almost 2-fold and over 3-fold more than those projected in moderate- and low-emission scenarios. Our results reveal the vulnerabilities and inequalities of children for the excess burden of anemia due to climate warming and highlight the importance of climate mitigation and adaptation strategies in LMICs.

## Graphical abstract

**Graphical abstract:**
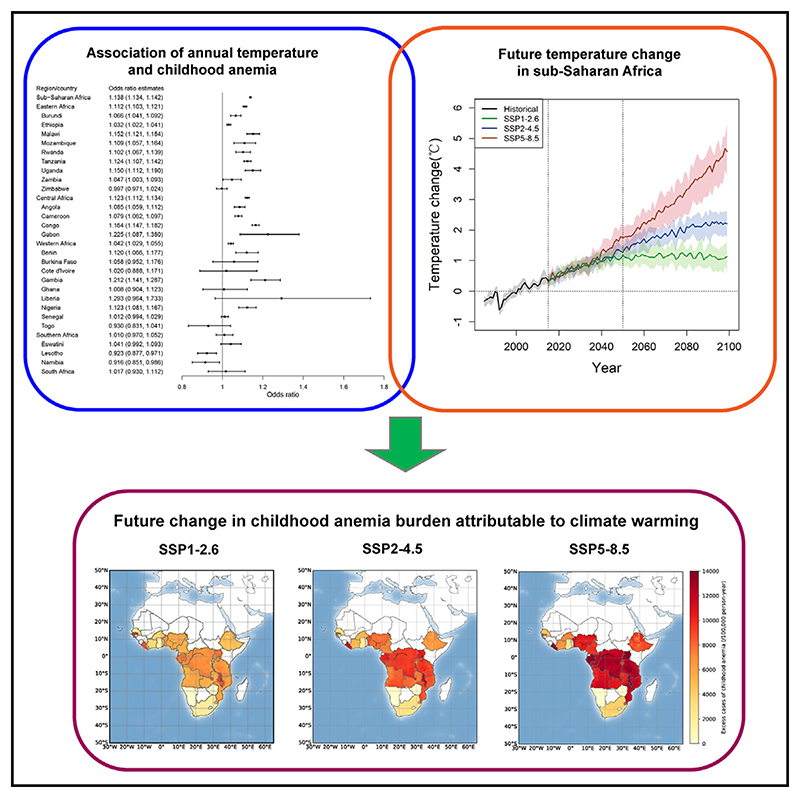

## Introduction

Childhood anemia constitutes a global public health problem, especially in low- and middle-income countries (LMICs).^1^ Based on estimates from the World Health Organization (WHO), 269.4 million children under 5 years suffered from anemia globally in 2020, and 38.0% of the cases were from sub-Saharan Africa.^2,3^ Children with anemia are at high risk of severe adverse health outcomes including cognitive or behavioral impairment,^4^ poor growth and development,^5^ and even death.^6^

The causes of childhood anemia are multi-factorial and inter-related in complex ways. The etiology of anemia could be related to proximate determinants (e.g., food insecurity, contaminated water, and sanitation problems), as well as some immediate causes, including nutritional deficiencies (iron, vitamins A and B12, etc.), infection (e.g., soil-transmitted helminth infection, schistosomiasis, and malaria), systemic inflammation, and genetic hemoglobin (Hb) disorders.^7^ In addition, epidemiological evidence indicated that environmental factors, such as high ambient temperature, may have some relationships with Hb concentration and anemia status in children.^8^ However, the possible roles of climate factors have rarely been considered in designing, monitoring, prevention, and control programs of anemia.^9,10^

Climate change has become the largest global threat for human health in the 21st century.^11^ Compared with recent decades (1995–2014), global surface air temperature is likely to increase by 2.4°C–4.8°C by the end of the 21st century with the assumption of unrestricted carbon emission, beyond the 2°C global temperature target of the Paris Agreement.^12^ Sub-Saharan Africa is predicted to warm faster than the global average during the 21st century.^13^ In addition, climate change is expected to disproportionately affect vulnerable groups (such as children under 5 years) and further exacerbate existing health inequities.^14^ LMICs, which produce the least greenhouse gas emissions, have far less capability to adapt to climate change.^15^ Consequently, vulnerable populations in LMICs will suffer from more health threats than those in high-income countries.^16^ Climate change is expected to reduce agricultural production,^17^ alter micronutrient content of food crops,^18^ and cause food insecurity. On the other hand, high ambient temperatures may shorten the breeding and development of mosquito, as well as the incubation period for Plasmodium parasites,^19^ which could increase the transmission of malaria.^20^ Malaria parasites could infect red blood cells and cause them to rupture (a process known as hemolysis), resulting in the spread of infectious anemia in children.^21^ A warming climate could thus lead to nutritional iron deficiency and susceptibility of parasitic infections, contributing to the development of anemia in children. However, few studies have directly linked ambient temperature with childhood anemia, and it remains poorly understood how the burden of childhood anemia will vary in a warming climate.^22^

To understand the impacts of global warming on childhood anemia in LMICs, we first explored the association of ambient temperature with the prevalence of childhood anemia across sub-Saharan Africa and then projected excess cases of childhood anemia attributable to global warming under selected climate change scenarios by the end of 21st century. We found that each 1°C increment in annual temperature was associated with increased odds of anemia (odd ratio = 1.138, 95% confidence interval: 1.134–1.142) in children. Compared with the baseline period (1985–2014), the excess childhood anemia cases attributable to climate change would increase by 7,597 (95% confidence interval [CI]: 4,133–10,894) per 100,000 person-years under a high-emission scenario in the 2090s, which would be almost 2-fold and over 3-fold more than those projected in moderate- and low-emission scenarios (Table S11). The results reveal the vulnerabilities and inequalities of children for the excess burden of anemia due to climate warming and highlight the importance of climate mitigation and adaptation strategies in LMICs.

## Results

### Descriptive data

**Figure 1:**
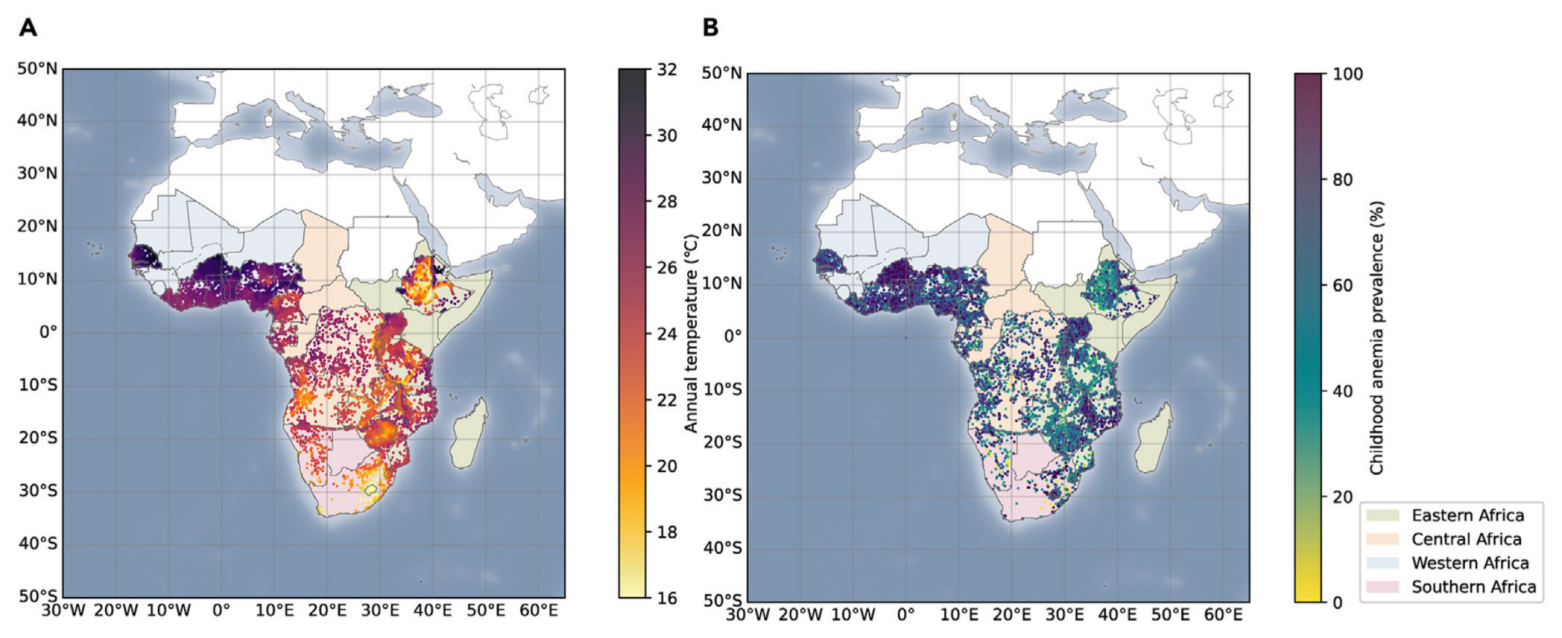
Temperature exposure and prevalence of childhood anemia in sub-Saharan Africa, 2003–2020 (A–B) (A) Annual temperature exposure for studied clusters. (B) Prevalence of childhood anemia for studied clusters in sub-Saharan Africa.

This study includes a total of 275,377 children under 5 years old from 26 sub-Saharan African countries between 2003 and 2020. The overall prevalence of childhood anemia is 63.76% (Table 1). Of anemia in different degrees, the prevalence of moderate anemia is highest (35.34%), followed by mild anemia (25.06%) and severe anemia (3.47%) (Table S1). Spatial distribution of childhood anemic cases and annual mean temperature levels across the sub-Saharan Africa are shown in Figure 1. Specifically, anemia prevalence is higher in Western Africa, Central Africa, and several subregions in Eastern Africa, where higher annual temperatures are also recorded. Southern Africa has a lower anemia prevalence and lower temperature. At the country level, annual average temperatures range from 16.3°C in Lesotho to 28.8°C in Burkina Faso (Table 1). Characteristics of the study population are summarized in Table S2. The mean age of children was 2.11 (±1.33) years, and 49.6% (N = 136,575) were girls.

### Baseline temperature-anemia association

**Figure 2:**
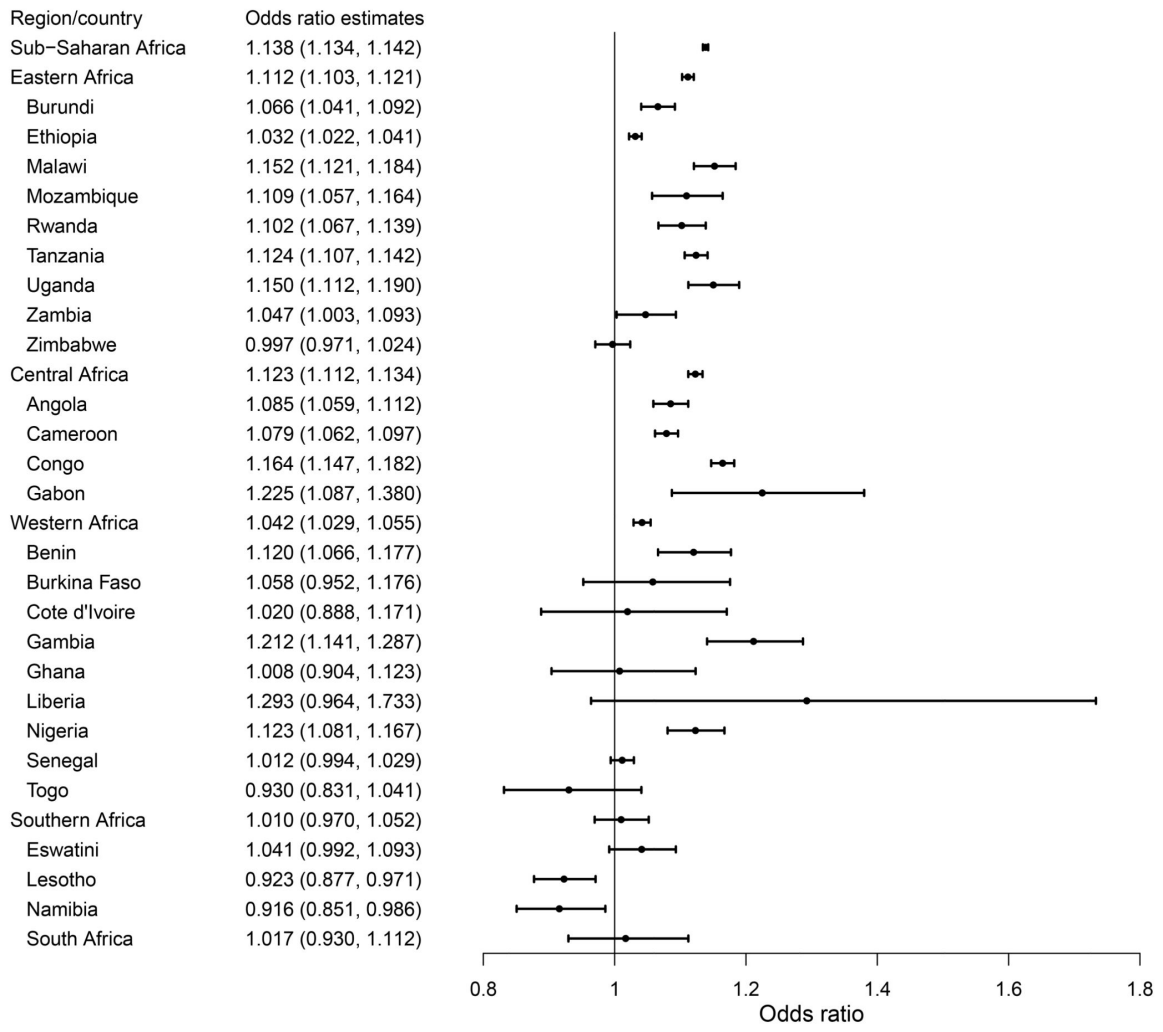
Odds ratios (95% confidence intervals) of childhood anemia per 1°C increase in annual temperature, classified by different regions or countries of sub-Saharan Africa

Figure 2 shows the odds ratio (OR) estimates for the associations between annual mean temperature and childhood anemia prevalence at the region and country levels. The OR of total anemia is 1.138 (95% CI: 1.134–1.142), associated with each 1°C increment in annual mean temperature. Regionally, there are higher ORs in Eastern Africa, Central Africa, and Western Africa than in Southern Africa. For specific countries, Gabon has the largest OR, while the estimates in most Southern African countries are not statistically significant. Specifically, for a 1°C increment in annual mean temperature, the increase in the prevalence of severe anemia (OR = 1.259, 95% CI: 1.245–1.273) is the largest, followed by moderate anemia (OR = 1.173, 95% CI: 1.169–1.178) and mild anemia (OR = 1.090, 95% CI: 1.085–1.095) (Table S3). Table S4 presents the corresponding risk ratio (RR) estimates in each country. The ORs of childhood anemia are larger when a longer average exposure period was used from 1 to 12 months preceding the interview day (Table S5). In addition, the main model exhibited a better model fit compared with the models in sensitivity analyses using shorter exposure periods (Table S6).

We further examined the possible mediation effects of childhood malnutrition and malaria infection. 11.40% and 9.74% of the associations between annual mean temperature and childhood anemia are estimated to be mediated by the prevalence of childhood malnutrition and malaria infection, respectively (Table S7).

In Figure S1, the shape of the exposure-response (E-R) relationship curve between annual mean temperatures and ORs of childhood anemia in sub-Saharan Africa is approximately linear within most of the temperature range, and the OR of childhood anemia consistently increases with higher annual temperature. There is no significant difference between linear and nonlinear models, supporting the empirically linear assumption.

In the sensitivity analysis, the main risk estimates are robust to the alternative removal of household factors (wealth level, floor material, roof material, water infrastructures, and sanitation infrastructures), country-level covariates, or annual cumulative precipitation (Table S8). When using a smaller lookback window (the lifetime exposure) for children under 1 year old, the results are also robust. The regional effect estimates are robust to the use of random effect meta-analysis based on the country-level ORs (Table S9).

### Temporal and spatial trends in temperature change

**Figure 3:**
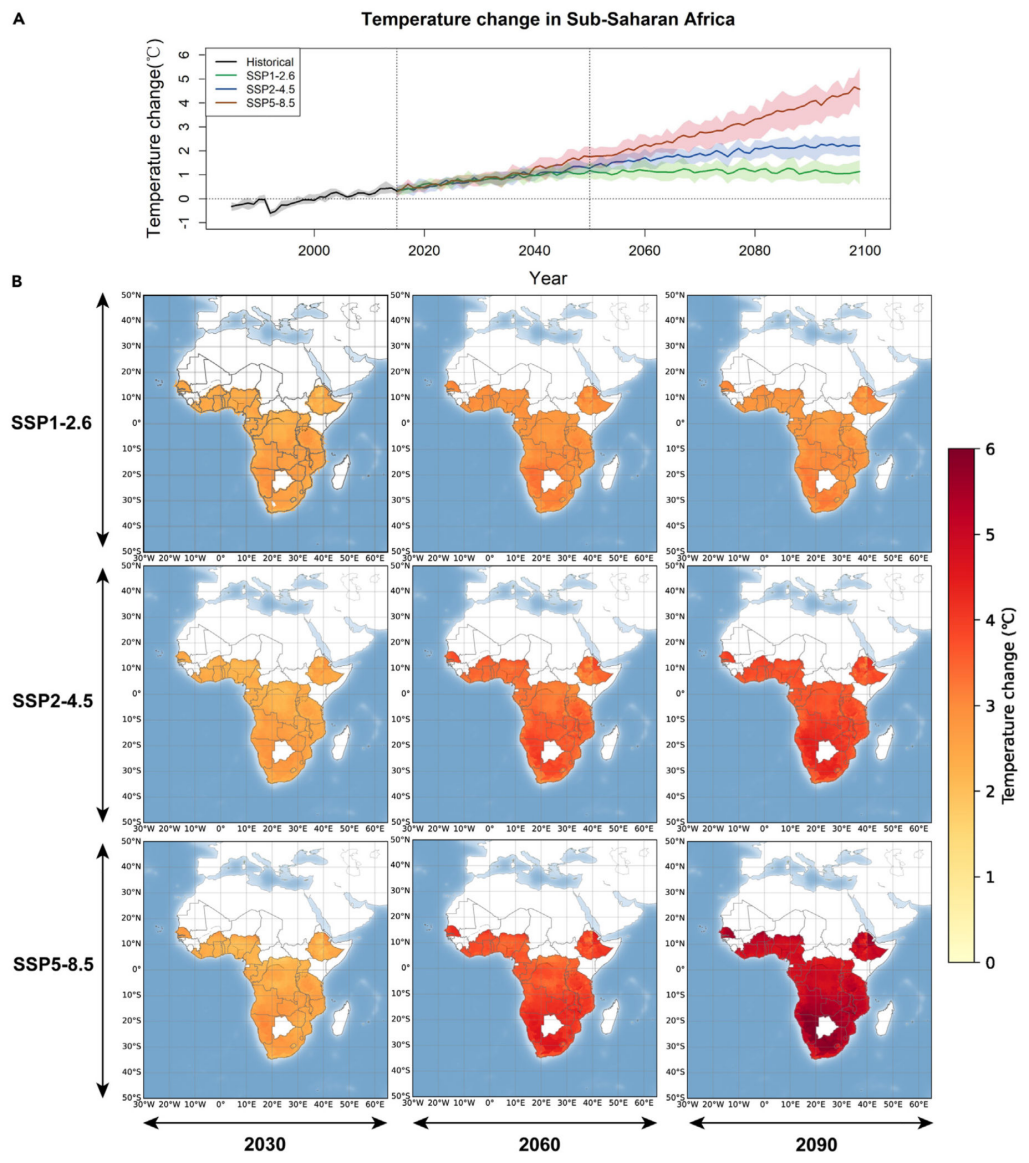
Projected temperature change from the baseline period (1985–2014) under different climate change scenarios in sub-Saharan Africa (A) Temporal trends of annual temperature change from the baseline period under three climate scenarios from 1985 to 2100. The shaded areas are the interquartile ranges of predicted temperatures from twenty general circulation models. (B) Spatial distribution of temperature change from the baseline period in cities in 2030, 2060, and 2090. SSP, shared socioeconomic pathway.

Figure 3 shows the trends of projected temperature change from the baseline year by region between 2015 and 2099 under three climate change scenarios (shared socioeconomic pathway [SSP]1-2.6, SSP2-4.5, and SSP5-8.5). We project a steep increase in annual mean temperatures across this century under the high-emission scenario (SSP5-8.5), while the increasing trends are modest under the SSP2-4.5 and SSP1-2.6 scenarios, which level off after the mid-21st century (Figure 3A). The annual temperatures show consistently increasing trends for all regions of sub-Saharan Africa, with the most prominent warming in Southern Africa (Figure 3B). For different climate change scenarios, the average annual temperatures in sub-Saharan Africa would increase by 4.0°C (95% CI: 3.1°C–4.6°C), 2.2°C (95% CI: 1.7°C–2.5°C), and 1.1°C (95% CI: 0.8°C–1.5°C) for the SSP5-8.5, SSP2-4.5, and SSP1-2.6 scenarios, respectively (Table S10). Regionally, Western Africa and Central Africa would experience higher temperature levels in the 2090s, whereas ambient temperature would increase more prominently in Southern Africa by the end of 21st century.

### Projected anemia burden due to climate change

**Figure 4:**
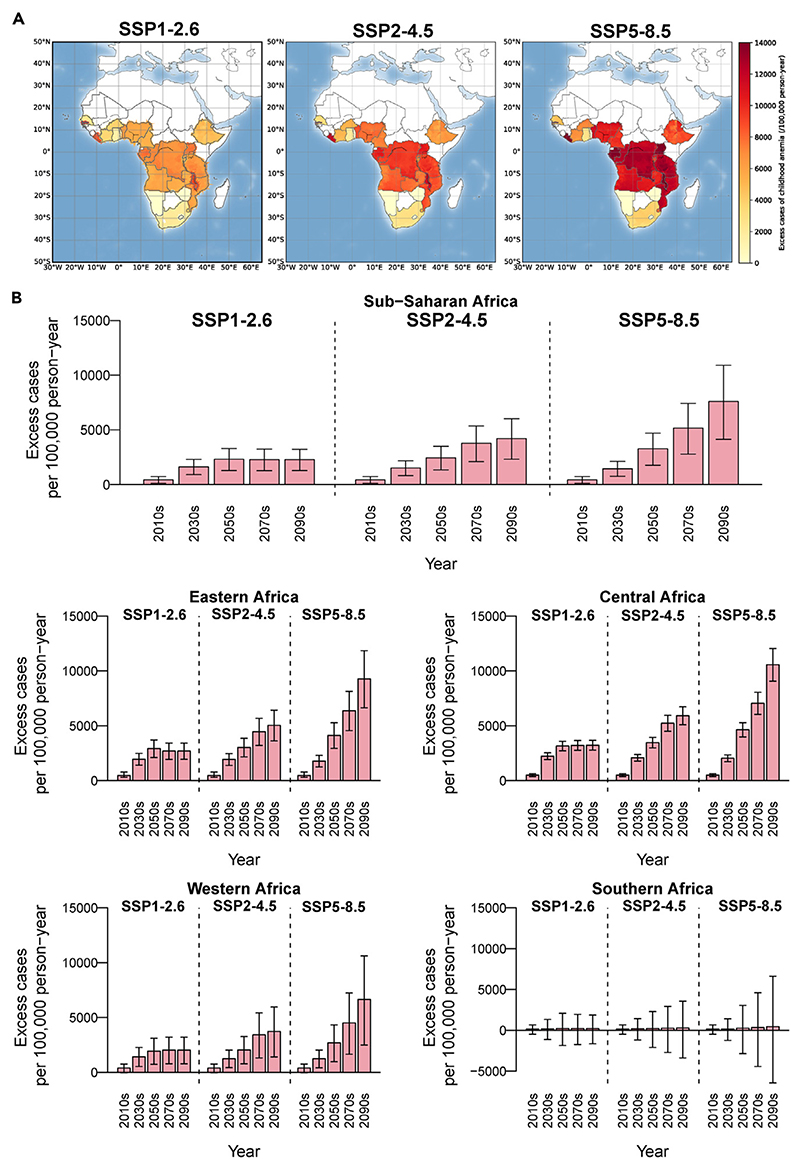
Projected excess cases of childhood anemia per 100,000 person-years attributable to climate warming in sub-Saharan Africa under different scenarios (A) Spatial distribution of excess cases of childhood anemia per 100,000 person-years in 2090s. (B) Projected excess cases of childhood anemia per 100,000 person-years attributable to climate warming (red bar) by year, country, and scenario. The black vertical lines represent the 95% empirical confidence intervals. SSP, shared socioeconomic pathway.

Figure 4 illustrates excess cases of childhood anemia per 100,000 person-years attributable to climate change in sub-Saharan Africa under three climate change scenarios (SSP1-2.6, SSP2-4.5, and SSP5-8.5). The spatial distributions of the projected disease burden are similar for the three scenarios, but the magnitude of future changes in burden differ considerably (Figure 4A). Under the assumption of no changes in population, anemia patterns, or climate adaption, there are increasing excess cases of childhood anemia in the projected period (2015–2099) in sub-Saharan Africa compared with the baseline (1985–2014) (Figure 4B). The increasing trends are steeper under the SSP5-8.5 scenario, whereas the trends are modest under SSP1-2.6 and become flat after the middle of this century under SSP2-4.5. Compared with the baseline period (1985–2014), the excess childhood anemia cases attributable to climate change would increase by 7,597 (95% CI: 4,133–10,894) per 100,000 person-years under the SSP5-8.5 scenario in the 2090s, which would be almost 2-fold and over 3-fold more than those in the SSP2-4.5 (4,208, 95% CI: 2,311–6,015) and SSP1-2.6 (2,275, 95% CI: 1,277–3,226) scenarios (Table S11).

Figure 4 also reveals different increasing patterns across regions. The excess cases of childhood anemia would rise more prominently in Central Africa than in other regions under SSP5-8.5, whereas there are nonsignificant increments in Southern Africa (Figure 4A). Taking the SSP5-8.5 scenario as an example, there would be increments of 10,566 (95% CI: 9,062–12,045) cases per 100,000 person-years in Central Africa, followed by 9,276 (6,637–11,854) cases in Eastern Africa and 6,646 (2,485–10,619) cases in Western Africa by the end of the 21st century (Table S11).

When the grided future population of children under 5 years is included in the projection of childhood anemia burden, the changes of excess cases of childhood anemia related to climate warming are not significantly different from those derived from our main analyses under the assumption of no population change (see Tables S11 and S12).

## Discussion

This epidemiological investigation in 26 sub-Saharan African countries demonstrates increased risk of childhood anemia prevalence associated with higher ambient temperature. We found that childhood malnutrition and malaria infection could mediate an appreciable portion of the association between high annual mean temperature and childhood anemia, respectively. Our study provides the first projection on the future burden of childhood anemia posed by global warming in sub-Saharan Africa, which would increase significantly, especially under the SSP5-8.5 scenario. We also observed significant regional disparities for the impacts of global warming on childhood anemia, with the highest burden in Central Africa, followed by Western Africa and Eastern Africa.

Although no epidemiological studies directly linked childhood anemia with ambient temperature, there are some indirect population-based evidences supporting our findings. A previous cross-sectional study found that land surface temperature was inversely associated with mean Hb concentration in preschool-age children, a core biomarker of childhood anemia.^8^ However, another study conducted in four sub-Saharan African countries did not observe a significant association.^23^ We observed significant associations between warmer ambient temperatures and higher prevalence of childhood anemia in three sub-Saharan African regions (Eastern, Central, and Western Africa) wherein children in Central Africa suffered the highest temperature-related anemia risk. Previous studies indicated that Central Africa had less precipitation and more vulnerability to drought than other regions.^24,25^ High temperature and low precipitation may co-exacerbate the childhood anemia condition in drought-prone regions.^26,27^ On the other hand, a nonsignificant association was observed in Southern African counties, where annual temperature was appreciably lower than other regions. The exposure-relationship curve in this study supports that the regions with a lower annual temperature range will experience lower childhood anemia risk.

We calculated the excess cases of childhood anemia attributable to annual temperatures. The projected burden of childhood anemia would increase in the context of global warming, but the magnitude would be highly affected by the extent and timing of warming under different climate change scenarios. There is a steep increase in future temperature, accompanied by a drastic increase in excess cases of childhood anemia under SSP5-8.5, a scenario characterized by unrestricted greenhouse gas emissions, population growth, energy consumption, and excessive land use. In contrast, the burdens of childhood anemia would increase modestly or plateau in the middle of this century under SSP2-4.5 or stricter SSP1-2.6, which are in accordance with the respective trends of temperature change in two scenarios. The findings emphasize the importance of developing targeted climate mitigation strategies and adaptation strategies for preventing the exacerbation of childhood anemia status in a changing climate.

The impact of climate change on childhood anemia seems to be biologically plausible. Two major culprits of anemia, namely malnutrition and parasitic infections, are both impacted under global warming. Previous studies have suggested that children’s nutritional status and susceptibility to pathogen infections may be affected by climate change through several pathways. First, vulnerability of agriculture to climate change increases the risk of child malnutrition. The combination of rising temperature and increasing atmospheric carbon dioxide may lead to child malnutrition and micronutrient deficits (such as iron deficiency) by attenuating agricultural productivity and crops’ nutrient content.^28,29^ Second, heat stress could reduce appetite and further result in malnutrition because of physiological adaption to high temperatures.^30^ Third, higher temperatures will promote the transmission of parasites and increase the risk of malaria infection among vulnerable populations, consequently increasing malaria-related anemia in a warmer climate.^31^ Furthermore, our analysis indicated that childhood malnutrition and malaria infection would partly mediate the impacts of high ambient temperature on childhood anemia, strengthening the biological plausibility of our epidemiological findings.^32–35^ High-temperature exposure could also induce a series of pro-inflammatory responses^36^ and thus disturb iron metabolism and red blood cell production, potentially increasing the risk of childhood anemia.^37,38^ In addition, high temperatures accompanied by drought can also lead to water scarcity, and the resulting poor water, sanitation, and hygiene conditions may lead to environmental enteric dysfunction, an underlying cause of anemia in children.^39,40^

We applied different lengths of exposure periods in exploring the association between ambient temperature and prevalence of childhood anemia and found that the association became stronger when a longer exposure period was used. The short-term impacts of a flood on infant mortality could be interpreted by several pathways. According to previous studies, relative shorter-term high-temperature exposure (1–3 months) may be linked with infectious diseases that mainly contribute to infective anemia,^41^ whereas longer-term exposure (e.g., annual temperature) may be linked with water resource, agricultural productivity, and malnutrition and, consequently, cause nutritional anemia.^42^

Although no previous studies predicted the childhood anemia burden in the context of global warming, our findings were also supported by previous projections on parasitic infections and malnutrition burden due to future climate change.^31,43,44^ For example, climate change would have a considerable impact on rates of severe stunting, which was estimated to increase by 31%, 36%, and 55% in Central, Western, and Eastern Africa in 2050, respectively.^45^ Similarly, Jesse et al. reported a 37% increase in the prevalence of child wasting associated with high temperature in Western Africa, while climate change was predicted to result in a 25% increase in the proportion of children with wasting in Eastern Africa and Central Africa by 2100 under the high-emission scenario.^44^ Although heterogeneity exists in study period, high-risk region, and disease burden estimates between our projections and previous studies, we found a similar temporal trend of increasing burden of these diseases for children in sub-Saharan Africa. Moreover, epidemic belt expansion and increased population at risk for malaria were reported under multiple climate change sceneries. The population at risk of malaria would increase up to about 736 million additional people by 2070 in the scenario of RCP8.5-SSP2, which may also lead to an increased burden of malaria-related anemia.^31^ However, temperature-related burdens of childhood anemia need to be fully captured in broader LMICs with unified analytical frameworks.

Our study findings have notable public health implications. First, we projected that temperature-related excess cases of childhood anemia would significantly increase under future climate change, especially under the high-emission scenario. Considering the challenges posed by global warming, we urgently need multi-sectoral collaborations to address long-term policies and plans to reduce anthropogenic carbon emissions to slow down the pace of global warming. Second, climate change disproportionally affects the populations in LMICs and vulnerable groups, adding threats to environmental equity and justice.^16^ Their vulnerabilities to climate change stress the urgency to implement measures to mitigate climate change hazards, including the establishment of high-temperature alerting systems,^46^ the effective allocation of medical resources,^47^ etc. Third, our results further suggested that preschool-aged children in LMICs suffered unevenly from the impacts of climate change on anemia.^48^ Local governments need to develop sophisticated protective strategies for this vulnerable group, for example, increasing popularization of air conditioning and indoor ventilation,^49^ improving housing conditions with thermal insulation materials,^33^ and advocating for the use of mosquito nets and iron supplementation in high-temperature regions.^34,35^

Several limitations of this study should be acknowledged. First, the E-R associations between annual temperature and childhood anemia were obtained through a cross-sectional study design, which attenuated the causality for our findings. Second, we projected childhood anemia burden under the assumption of no changes in anemia patterns and climate adaptation, so our prediction may represent the upper limit of the contribution of climate warming to anemia prevalence.^50^ Third, our dataset only included LMICs in tropical and sub-tropical regions, so caution should be taken when extrapolating our results to other regions.

## Conclusions

This multi-country study in sub-Saharan Africa demonstrates that higher environmental temperature could increase the prevalence of childhood anemia. Global warming would further significantly increase the burden of childhood anemia in this area. These results reveal the vulnerabilities and inequalities of children in LMICs in suffering from anemia under global warming and highlight the importance of mitigation and adaptation strategies of climate change, especially for vulnerable subgroups in climate-sensitive regions.

## Experimental Procedures

### Resource availability

#### Lead contact

Requests for further information and resources should be directed to the lead contact, Haidong Kan (kanh@fudan.edu.cn).

#### Materials availability

No materials were used in this study.

### Data sources

The DHS program collected health, behavior, and sociodemographic data routinely (about every 5 years) in more than 90 countries, covering a series of topics such as maternal and child health, malaria, domestic violence, and environmental health.^1^ In DHS survey, a “cluster” is a group of adjacent households and serves as the primary sampling unit in sampling procedure. Using a stratified two-stage cluster sampling design, clusters are randomly selected from the areas stratified by geographic region and by urban/rural area within each region, while the households are randomly selected from each cluster. From eligible households, all ever-married women of reproductive ages 15–49 were interviewed by trained fieldwork staff, and data of children under 5 years were collected from all clusters of the countries.

We included the following variables of children: age (<2 and 2–5 years), gender (male and female), body mass index (BMI), and result of malaria with rapid diagnostic test (RDT). Mother’s age (15–19, 20–29, 30–39, and 40–49 years) and education level (no education, primary, secondary, and higher) are adjusted for as indicators of socioeconomic status. We further controlled household characteristics including type of residence (urban and rural); one or more children in the household (singleton and multiple); type of water infrastructures (surface water, well, piped/tap, and others); type of sanitation infrastructures (no facility, latrine, flush toilet, and others); materials of floor (natural, rudimentary, finished, and others); materials of roof (natural, rudimentary, finished, and others); and wealth level. We calculated the scores of household wealth index using a linear principal-component analysis (PCA) based on the data of household assets including electricity, car, television, refrigerator, bicycle, scooter, mobile telephone, and cooking fuel (results of the PCA are shown in Table S13). Each survey separates interviewed households into five wealth quintiles (poorest, poorer, middle, richer, and richest) to characterize their wealth level.

### Health outcome

In all surveys, children aged 5 years or younger in the eligible households were tested for anemia. Hb concentrations in children were measured by finger- or heel-prick blood specimens using a portable Hemo Cue autoanalyzer.^51^ In accordance with the WHO definition of anemia in children under 5 years, a child was considered anemic if his or her altitude-adjusted Hb level was less than 11 g/dL. An Hb level below 7.0 g/dL was classified as severe anemia, a level between 7.1 and 9.9 g/dL was classified as moderate anemia, and a level between 10.0 and 10.9 g/dL was classified as mild anemia.^52^ Informed consent to participate in DHS interviews and biomarker tests was obtained orally from parents or guardians.

### Historical meteorological parameters and population data

Historical data of ambient temperature and precipitation were derived from ERA-5, a global reanalysis dataset on latitude-longitude grids at a resolution of approximately 0.25° × 0.25° and up to 1 h frequency, which was produced by the European Center for Medium Range Weather Forecasts (ECMWF).^53^ We assigned daily mean temperature and daily cumulative precipitation based on available geocoded coordinates for their clusters from January 1, 2003, to December 31, 2020. Then, we computed the annual average temperature and annual cumulative precipitation for each child under 5 years over the 365 days (1 year) prior to the interview day.

We obtained country-specific population data of under 5-year-old children for the survey year from the United Nations International Children’s Emergency Fund (UNICEF) Data Warehouse. Prevalence of anemia in children aged 6–59 months in the corresponding country and survey year was obtained from the Global Health Observatory project of the WHO. The crop production index and gross domestic product per capita for each sub-Saharan country in the survey year were derived from the World Bank.

### Future meteorological parameters

Future time series data of daily mean temperature for various climate change scenarios were derived from the latest internationally coordinated Coupled Model Intercomparison Project, sixth phase (CMIP6).^12^ The projections of global climate change were assessed based on the SSPs, which include five common scenarios (i.e., SSP1-1.9, SSP1-2.6, SSP2-4.5, SSP3-7.0, and SSP5-8.5). These scenarios correspond to the increasing trajectories of atmospheric greenhouse gas concentration and describe a range of warming in global climate from mild (SSP1-1.9) to extreme (SSP5-8.5). We selected three most common scenarios and the baseline period (1995–2014) in accordance with previous projection studies.^12,54^ Compared to the baseline period, global surface air temperature is likely to increase 2.4°C–4.8°C in the high-emission scenario (SSP5-8.5) over the period 2081-2100 and by 0.5°C–1.5°C and 1.2°C–2.6°C for the low- and moderate-emission scenarios (SSP1-2.6 and SSP2-4.5), respectively.^12^ Finally, we extracted daily temperature data during the baseline period and the projection period (2015–2099) from 20 global climate model (GCM) datasets for historical and future temperature simulations in various climate change scenarios (Table S14).

Temperature data of GCM outputs were interpolated statistically to a geographical grid of a 1.0° 3 1.0° resolution using a bilinear interpolation method and were then transformed into city-level estimates by spatially averaging the gridded data within each sub-Saharan African country. Cities and boundaries were defined based on the Database of Global Administrative Areas v.4.1 (https://gadm.org/).^55^ We downloaded data of the included 26 countries and extracted the boundaries at administrative level 2, which was deemed as the city level in this analysis. However, the projected temperature series derived from different GCMs may result in nonnegligible bias when data were applied to fitting the association between temperature and health outcomes quantified by ERA-5 reanalysis data. Therefore, we extracted the modeled daily temperature series for each grid in the studied country during 1985–2099 and further corrected the modeled temperatures with data in corresponding grids from ERA-5 reanalysis series by using an additive scaling method for all GCMs.^56^

### Future population data

We obtained predicted grided population size data under the SSP1 (sustainability), SSP2 (middle of the road), and SSP5 (fossil-fueled development) scenarios from National Aeronautics and Space Administration (NASA) Socioeconomic Data and Applications Center (SEDAC).^57^ The database provides global urban, rural, and total population base year and projection grids at a resolution of 1 km (about 30 arc seconds) based on the SSPs.

We further obtained projected total population and population for children aged under 5 years at the country level, using the SSP Database v.2.0, under the same scenarios above.^58^ Due to the unavailability of population age structure projections for all countries, we applied country-level projections to each location in 10 year intervals from 2020 to 2100.

We then calculated the proportion of children under 5 years in a future year during 2020–2100 by dividing the projected age-group-specific population for that year by the total population projected for the same year. Finally, we utilized the proportion of children under 5 years at the country level and applied it to the predicted population grid for the corresponding year and SSP in order to acquire the population grid of children under 5 years for the future.

### Statistical analyses

#### Temperature-anemia association

Because previous studies have found associations between higher temperatures and increases in common anemia risk factors, such as malnutrition and malaria infection,^59,60^ we hypothesized a linear E-R relationship between annual mean temperature and the prevalence of childhood anemia and only explored the impact of temperature increase in sub-Saharan Africa. Then, mixed-effect multi-variable logistic regression models were applied to estimate the associations between annual mean temperature and anemic status (binary variable) of children at the individual level. Based on literature about the risk factor of childhood anemia,^61,62^ we adjusted for the following covariates in the main model: age, gender, BMI, insecticide-treated net use, type of residence, one or more children in the household, mother’s age and education level, and household wealth level. We controlled for the type of WASH (water, sanitation, and hygiene) infrastructures and annual cumulative precipitation, which may affect the spread of infectious diseases in a population. Materials of roof and floor were also adjusted, which could modify the effect of ambient temperature on the residents inside the house. To better isolate the effect of temperature increase, we control for decadal mean temperature and random effects for city in the main model. We also included other country-level covariates, including crop product index and gross domestic product per capita in the model. Finally, we controlled survey month and year to adjust for seasonality and long-term trends, respectively. The effect of ambient temperature on anemia status was presented as the OR and its 95% CIs of childhood anemia prevalence associated with a 1°C increment in mean temperature over the past year.

To allow for the calculation of excess cases, we applied the following formula (Equation 1) to estimate the relative risks (RRs) (and 95% CI) in each sub-Saharan African country.^63^

(Equation 1) $$RR_{c}=\frac{OR_{c}}{1-\;Rate{\;}_{c}+\;Rate{\;}_{c}\times OR_{c}},$$

where *RR_c_* is the estimated RR (and 95% CI) of childhood anemia incidence associated with a 1°C increment of annual mean temperature in each country and *Rate_c_* is the country-specific prevalence of childhood anemia over the corresponding survey year.

In addition, we examined whether the effect estimates vary by different exposure periods. Specifically, we calculated the average temperatures during the past 1, 3, 6, and 9 months preceding the interview day and then reperformed the main analysis for the association between temperature and childhood anemia prevalence in each exposure period.

Furthermore, we conducted a causal mediation analysis to examine the possible roles of childhood malnutrition and malaria infection on the association between high-temperature exposure and childhood anemia. Childhood malnutrition was defined as a Kaup index (BMI in children) <15 kg/m^−2^.^64^ Data on childhood malaria infection were collected by RDT in the DHS survey. Specifically, we fit the main model and the adjusted model (i.e., the main model adjusted by the presence of malnutrition or malaria infection) to calculate the total effect (TE) and the direct effect (DE), respectively. The indirect effect (IE) of the mediator was calculated by the association coefficient of high-temperature exposure in the main model (TE) minus the corresponding coefficient in the mediator-adjusted model (DE). Then, the percentage of the mediation effect was calculated as the proportion of the IE in the TE from the main model. The empirical 95% CIs for the IE were estimated using a bootstrap resampling with 1,000 samples.

To examine the shape of temperature-anemia association and test the nonlinearity of the curve, we flexibly depicted the E-R relationship curve between annual mean temperature and childhood anemia prevalence using the generalized additive model with a natural cubic spline of 3 degrees of freedom for temperature and covariates. We used F tests in analysis of variance (ANOVA) to test differences between the linear model and the nonlinear model.

We tested the robustness of temperature-anemia association in three sensitivity analyses. First, we fit three separate models based on the main model: (1) leaving out the household factors (wealth level, floor material, roof material, water infrastructures, and sanitation infrastructures); (2) removing countrylevel covariates; and (3) leaving out annual cumulative precipitation. Second, we conducted a random effect meta-analysis based on the country-level ORs to derive the regional effect estimates. Third, we conducted a sensitivity analysis by adding gridded future population of children under 5 years in the projection of anemia burden prevalence.

#### Future changes in anemia burden due to climate warming

To reveal the potential impact of climate change on anemia, we projected changes in childhood anemia burden due to warming temperature, assuming no changes of population, anemia patterns, or climate adaption over the projection period (2015–2099). Under the assumption of linear temperature-anemia association, we calculated the historical and future numbers of excess cases of childhood anemia due to annual mean temperature increase as

(Equation 2) $$\text{Excess}\;\text{cases}=\;Pop{\;}_{c}\times \;Rate{\;}_{c}\times ERC_{c}\times \text{Δ}T,$$

where *Pop_c_* is the size of population under 5 years old at the city level in each country; *Rate*_c_ represents the baseline annual prevalence of childhood anemia in each country during the survey year; *ERC_c_* represents the country-specific percentage change in the risk of childhood anemia for a 1°C increment in temperature; and *ΔT* indicates the predicted changes in future annual temperature for each city, relative to the average baseline temperature between 1985 and 2014. The population size at baseline and projected temperatures was aggregated by grid-level data at the city level. Finally, we calculated the childhood anemia burden attributable to climate change as the proportion of excess childhood anemia cases related to temperature increase in the total numbers of children under 5 years at baseline in each city, multiplying 100,000 personyears. The excess cases were aggregated by country, decade, and scenario and were then used to calculate the future changes in childhood anemia burden due to climate warming. We then obtained total attributable burden of childhood anemia by taking the average value of the attributable burden calculated by all 20 GCMs. The empirical CIs (eCIs) were calculated by generating 1,000 samples of the coefficients through Monte Carlo simulations, assuming a normal distribution for the estimated coefficients, to quantify the uncertainty in estimations of the E-R relationships and the variability in temperature projections for each of the twenty GCMs. We finally obtained eCIs corresponding to the 2.5^th^ and 97.5^th^ percentiles of the distribution of the results across coefficients and 20 GCMs.

All statistical analyses were conducted in the R software (v.4.0.3, R Project for Statistical Computing) with the “hyfo” package for bias-correction process, the “lm4e” package for mixed effects multi-variable logistic models, and the “meta” package for meta-regression analyses. All statistical tests were two-sided, and a p value <0.05 was considered statistically significant.

## Supplementary Material

Supplementary material

## Figures and Tables

**Table 1:** Summary statistics for the country-specific distribution of children under 5 years and annual temperature

Region/country	N	Percentage (%)	Anemia prevalence (%)	Temperature (°C)		
				Mean ± SD	Min	Max
Eastern Africa	103,628	37.63	55.36	21.7 ± 2.6	12.6	32.6
Burundi	16,974	6.16	53.31	21.0 ± 1.4	17.7	26.1
Ethiopia	19,529	7.09	55.01	21.3 ±4.2	12.6	32.6
Malawi	10,992	3.99	65.59	22.2 ±1.7	16.9	26.6
Mozambique	4,355	1.58	66.25	23.6 ± 1.5	18.4	26.7
Rwanda	10,636	3.86	42.51	19.5 ± 1.7	15.6	25.7
Tanzania	14,766	5.36	56.39	23.4 ±2.4	15.4	27.1
Uganda	7,842	2.85	58.70	23.0 ± 1.8	15.1	27.1
Zambia	7,722	2.80	58.43	21.9 ± 1.3	19.0	26.1
Zimbabwe	10,812	3.93	51.06	21.3 ± 1.8	17.4	27.0
Central Africa	58,407	21.21	63.94	24.1 ±2.3	16.2	29.8
Angola	11,198	4.07	64.99	22.8 ± 1.9	18.6	25.9
Cameroon	23,502	8.53	62.24	24.7 ±2.6	18.7	29.8
Congo Democratic Republic	20,692	7.51	65.32	24.1 ±2.1	16.2	27.3
Gabon	3,015	1.09	63.81	25.0 ±0.7	22.6	26.1
Western Africa	103,586	37.62	73.42	27.7 ± 1.4	19.8	31.8
Benin	17,652	6.41	67.14	27.4 ±0.8	25.5	30.1
Burkina Faso	17,532	6.37	89.25	28.8 ±0.6	26.8	30.7
Cote d’Ivoire	5,560	2.02	75.29	26.3 ±0.6	24.2	28.0
Gambia	3,256	1.18	53.38	27.7 ± 1.7	25.1	30.3
Ghana	7,549	2.74	74.84	27.0 ± 1.1	25.1	29.5
Liberia	2,149	0.78	71.20	25.9 ±0.4	24.6	26.6
Nigeria	10,168	3.69	68.79	27.0 ± 1.2	19.8	29.8
Senegal	36,840	13.38	71.68	28.0 ± 1.7	23.7	31.8
Togo	2,880	1.05	71.01	27.2 ±1.0	24.8	29.4
Southern Africa	9,756	3.54	49.36	17.0 ±4.6	12.8	25.9
Eswatini	3,546	1.29	43.99	19.1 ±2.1	15.4	25.8
Lesotho	3,876	1.41	51.24	16.3 ±2.8	12.8	25.7
Namibia	1,529	0.56	51.21	22.4 ±2.0	15.9	25.9
South Africa	805	0.29	60.50	19.4 ±2.0	13.8	24.1
Total	275,377	100.00	63.76	24.4 ±3.7	12.6	32.6

SD, standard deviation; Min, minimum; Max, maximum.

## Data Availability

The data and code used in this analysis can be accessed online. The details of databases are listed below: (1) data of Demographic and Health Surveys (DHS) conducted in sub-Saharan Africa (https://dhsprogram.com/); (2) historical data of ambient temperature and precipitation (https://cds.climate.copernicus.eu/); (3) country-specific population data (https://data.unicef.org/dv_index/) and anemia prevalence (https://www.who.int/data/gho/) of children under 5 years old; (4) country-specific crop production index and gross domestic product per capita (https://data.worldbank.org/indicator/); (5) future temperature exposure under different scenarios (https://esgf-node.llnl.gov/search/cmip6/); and (6) future population-size data (https://sedac.ciesin.columbia.edu/data/collection/gpw-v4) and the proportion of children under 5 years (https://tntcat.iiasa.ac.at/SspDb/) under different scenarios. The details of codes are listed below: source code for temperature-anemia association and future childhood anemia burden projection (https://github.com/YixiangZhu/Anemia-temperature/). Any additional information required to reanalyze the data reported in this paper are available from the lead contact upon request.

## In brief

Zhu et al. provide the evidence that higher annual temperature was associated with increased prevalence of childhood anemia. The burden of childhood anemia attributable to climate warming would increase significantly under a highemission scenario by the end of 21st century. The results reveal the vulnerabilities and inequalities of children for the anemia burden due to climate warming and highlight the importance of climate mitigation and adaptation strategies in low- and middle-income countries.

## Highlights

- Higher annual temperature was associated with increased prevalence of childhood anemia
- Associations could be partly mediated by childhood malnutrition and malaria infection
- Childhood anemia burden due to climate warming may increase significantly in the future

## Science For Society

Anemia, a condition in which hemoglobin concentration and/or red blood cell numbers are lower than normal, can lead to fatigue, weakness, dizziness, and shortness of breath. Affecting ~270 million children under 5 years in 2020, anemia is a serious global public health problem and is particularly prevalent in sub-Saharan Africa, where 38% of cases originate. Anemia may be caused by several factors, which are considered in monitoring, prevention, and control programs for anemia. However, climatic factors such as rising temperatures are rarely considered, which may lead to an underestimation of the disease burden and a gap in the protection of children’s health as the climate changes. This study reveals significant associations between higher annual temperatures and increased prevalence of childhood anemia, and results indicate that the disease burden attributable to climate warming could increase significantly under a high-emission scenario by the end of the 21st century. Governments in low- and middle-income countries should heed these findings and implement comprehensive climate mitigation and adaptation strategies to help children face the growing risks of climate change.
