# Supplementary material for: Global warming may significantly increase childhood anemia burden in sub-Saharan Africa
Source: One Earth. Author manuscript; Available in PMC 2023 Oct 30. (PMC7615260; doi:10.1016/j.oneear.2023.09.003)
Supplement: Supplementary material [file EMS189975-supplement-Supplementary_material.pdf]

One Earth, Volume *6*

Supplemental information

Global warming may significantly increase childhood anemia burden in sub-Saharan Africa

Yixiang Zhu, Cheng He, Antonio Gasparrini, Ana Maria Vicedo-Cabrera, Cong Liu, Jovine Bachwenkizi, Lu Zhou, Yuexin Cheng, Lena Kan, Renjie Chen, and Haidong Kan

Table S1. Prevalence of childhood anemia in different level in 26 sub-Saharan African countries during 2003–2020.

Region/Country	Mild anemia	Moderate anemia	Severe anemia
Eastern Africa	24.57%	28.41%	2.38%
Burundi	24.21%	26.72%	2.37%
Ethiopia	22.50%	28.56%	3.95%
Malawi	25.32%	37.43%	2.85%
Mozambique	26.18%	36.69%	3.38%
Rwanda	22.21%	19.19%	1.11%
Tanzania	26.27%	28.57%	1.55%
Uganda	23.27%	32.10%	3.33%
Zambia	28.57%	28.39%	1.48%
Zimbabwe	25.59%	24.45%	1.03%
Central Africa	26.38%	34.44%	3.12%
Angola	30.20%	32.18%	2.61%
Cameroon	25.71%	33.73%	2.80%
Congo Democratic Republic	24.63%	36.73%	3.96%
Gabon	29.52%	32.54%	1.76%
Western Africa	24.92%	43.55%	4.96%
Benin	27.22%	36.70%	3.23%
Burkina Faso	18.24%	59.38%	11.64%
Cote d'Ivoire	24.93%	46.83%	3.53%
Gambia	24.72%	26.81%	1.84%
Ghana	23.30%	46.19%	5.35%
Liberia	28.11%	40.16%	2.93%
Nigeria	27.05%	38.59%	3.16%
Senegal	26.49%	41.38%	3.82%
Togo	25.87%	42.53%	2.60%
Southern Africa	23.85%	24.15%	1.36%
Eswatini	22.50%	20.53%	0.96%
Lesotho	24.41%	25.18%	1.65%
Namibia	24.66%	25.57%	0.98%
South Africa	25.59%	32.42%	2.48%
Total	25.06%	35.34%	3.47%

Table S2. Characteristics of the study population in different anemia level in 26 sub-Saharan African countries during 2003–2020.

Variables	Not anemia (N=99,795)	Mild anemia (N=69,013)	Moderate anemia (N=97,012)	Severe anemia (N=9,557)
Region				
Urban	31,897 (32.0%)	20,945 (30.3%)	24,714 (25.5%)	1,622 (17.0%)
Rural	67,898 (68.0%)	48,068 (69.7%)	72,298 (74.5%)	7,935 (83.0%)
Gender				
Male	48,152 (48.3%)	34,644 (50.2%)	50,787 (52.4%)	5,219 (54.6%)
Female	51,643 (51.7%)	34,369 (49.8%)	46,225 (47.6%)	4,338 (45.4%)
Age (y)				
<2	25,697 (25.7%)	25,821 (37.4%)	45,125 (46.5%)	4,973 (52.0%)
2-5	74,098 (74.3%)	43,192 (62.6%)	51,887 (53.5%)	4,584 (48.0%)
One or more children in the household				
Singleton	2,738 (2.7%)	1,983 (2.9%)	3,338 (3.4%)	445 (4.7%)
Multiple	97,057 (97.3%)	67,030 (97.1%)	93,674 (96.6%)	9,112 (95.3%)
Sleep under bed net last night				
Yes	62,545 (62.7%)	44,678 (64.7%)	62,480 (64.4%)	5,645 (59.1%)
No	37,250 (37.3%)	24,335 (35.3%)	34,532 (35.6%)	3,912 (40.9%)
Mother's age				
15-19	3418 (3.4%)	3240 (4.7%)	5897 (6.1%)	722 (7.6%)
20-29	47737 (47.8%)	34041 (49.3%)	48677 (50.2%)	4821 (50.4%)
30-39	38037 (38.1%)	25090 (36.4%)	33727 (34.8%)	3187 (33.3%)
40-49	10603 (10.6%)	6642 (9.6%)	8711 (9.0%)	827 (8.7%)
Mother's highest education				
No education	36438 (36.5%)	29468 (42.7%)	48648 (50.1%)	5969 (62.5%)
Primary	37508 (37.6%)	23754 (34.4%)	30905 (31.9%)	2527 (26.4%)
Secondary	23047 (23.1%)	14487 (21.0%)	16430 (16.9%)	1017 (10.6%)
Higher	2802 (2.8%)	1304 (1.9%)	1029 (1.1%)	44 (0.5%)
Water infrastructures				
Basic (surface water)	36,080 (36.2%)	23,316 (33.8%)	28,333 (29.2%)	2,072 (21.7%)

Intermediate (well)	34,166 (34.2%)	27,097 (39.3%)	43,687 (45.0%)	4,802 (50.2%)
Modern (piped/tap)	27,057 (27.1%)	16,785 (24.3%)	23,050 (23.8%)	2,523 (26.4%)
Others	2,492 (2.5%)	1,815 (2.6%)	1,942 (2.0%)	160 (1.7%)
Sanitation infrastructures				
Basic (no facility)	12,376 (12.4%)	8,066 (11.7%)	8,317 (8.6%)	411 (4.3%)
Intermediate (latrine)	65,057 (65.2%)	42,024 (60.9%)	55,602 (57.3%)	4,812 (50.4%)
Modern (flush toilet)	21,400 (21.4%)	18,235 (26.4%)	32,066 (33.1%)	4,245 (44.4%)
Others	962 (1.0%)	688 (1.0%)	1,027 (1.1%)	89 (0.9%)
Floor Material				
Natural	54,700 (54.8%)	37,380 (54.2%)	55,694 (57.4%)	6,295 (65.9%)
Rudimentary	650 (0.7%)	515 (0.7%)	736 (0.8%)	64 (0.7%)
Finished	44,078 (44.2%)	30,830 (44.7%)	40,319 (41.6%)	3,167 (33.1%)
Others	367 (0.4%)	288 (0.4%)	263 (0.3%)	31 (0.3%)
Roof Material				
Natural	33,077 (33.1%)	24,624 (35.7%)	40,904 (42.2%)	5,041 (52.7%)
Rudimentary	4,416 (4.4%)	2,840 (4.1%)	4,039 (4.2%)	462 (4.8%)
Finished	61,833 (62.0%)	41,136 (59.6%)	51,420 (53.0%)	3,968 (41.5%)
Others	469 (0.5%)	413 (0.6%)	649 (0.7%)	86 (0.9%)
Wealth level				
Poorest	21,463 (21.5%)	17,327 (25.1%)	27,145 (28%)	2,910 (30.4%)
Poorer	23,095 (23.1%)	16,445 (23.8%)	26,070 (26.9%)	3,236 (33.9%)
Middle	25,768 (25.8%)	16,801 (24.3%)	23,969 (24.7%)	2,304 (24.1%)
Richer	5,287 (5.3%)	3,639 (5.3%)	4,511 (4.6%)	331 (3.5%)
Richest	24,182 (24.2%)	14,801 (21.4%)	15,317 (15.8%)	776 (8.1%)

Table S3. Odds ratios (95% confidence intervals) of childhood anemia in different degrees per 1°C increase in annual temperature in different sub-Saharan African countries.

Country	Mild anemia	Moderate anemia	Severe anemia
Total	1.090 (1.085, 1.095)	1.173 (1.169, 1.178)	1.259 (1.245, 1.273)
Eastern Africa	1.080 (1.069, 1.090)	1.143 (1.131, 1.154)	1.142 (1.106, 1.178)
Burundi	1.017 (0.988, 1.046)	1.105 (1.073, 1.137)	1.161 (1.073, 1.257)
Ethiopia	1.024 (1.012, 1.035)	1.052 (1.041, 1.064)	1.048 (1.023, 1.074)
Malawi	1.130 (1.093, 1.168)	1.174 (1.137, 1.212)	1.140 (1.042, 1.247)
Mozambique	1.109 (1.046, 1.176)	1.108 (1.049, 1.171)	1.121 (0.981, 1.282)
Rwanda	1.047 (1.010, 1.087)	1.180 (1.126, 1.237)	1.090 (0.896, 1.326)
Tanzania	1.095 (1.075, 1.115)	1.147 (1.126, 1.169)	1.136 (1.062, 1.216)
Uganda	1.074 (1.033, 1.118)	1.193 (1.146, 1.243)	1.189 (1.080, 1.310)
Zambia	0.986 (0.936, 1.037)	1.078 (1.023, 1.135)	1.271 (1.081, 1.495)
Zimbabwe	0.992 (0.961, 1.023)	1.013 (0.980, 1.047)	1.017 (0.891, 1.160)
Central Africa	1.098 (1.086, 1.111)	1.139 (1.126, 1.152)	1.120 (1.088, 1.153)
Angola	1.075 (1.044, 1.107)	1.082 (1.051, 1.113)	1.043 (0.963, 1.130)
Cameroon	1.066 (1.046, 1.087)	1.095 (1.074, 1.116)	1.001 (0.948, 1.058)
Congo Democratic Republic	1.117 (1.097, 1.137)	1.191 (1.170, 1.213)	1.209 (1.158, 1.263)
Gabon	1.296 (1.121, 1.498)	1.200 (1.043, 1.379)	1.274 (0.811, 2.000)
Western Africa	1.024 (1.009, 1.039)	1.051 (1.036, 1.066)	1.133 (1.096, 1.171)
Benin	1.111 (1.047, 1.179)	1.128 (1.067, 1.193)	1.479 (1.310, 1.670)
Burkina Faso	1.030 (0.910, 1.166)	1.083 (0.972, 1.206)	1.295 (1.108, 1.513)
Cote d'Ivoire	0.991 (0.843, 1.166)	1.089 (0.939, 1.264)	1.070 (0.769, 1.488)
Gambia	1.111 (1.035, 1.192)	1.354 (1.256, 1.460)	1.485 (1.128, 1.953)
Ghana	0.973 (0.858, 1.104)	1.047 (0.928, 1.182)	0.933 (0.730, 1.193)
Liberia	1.221 (0.861, 1.732)	1.281 (0.925, 1.774)	3.460 (1.342, 8.919)
Nigeria	1.122 (1.072, 1.175)	1.136 (1.088, 1.187)	1.098 (0.997, 1.210)
Senegal	0.999 (0.979, 1.020)	1.034 (1.014, 1.055)	1.140 (1.081, 1.202)

Togo	0.989 (0.870, 1.125)	0.893 (0.792, 1.007)	0.783 (0.568, 1.079)
Southern Africa	1.010 (0.962, 1.060)	1.014 (0.962, 1.068)	1.029 (0.826, 1.281)
Eswatini	1.021 (0.966, 1.079)	1.041 (0.982, 1.103)	0.976 (0.759, 1.254)
Lesotho	0.938 (0.881, 0.998)	0.915 (0.859, 0.974)	0.831 (0.674, 1.025)
Namibia	0.909 (0.835, 0.990)	0.953 (0.872, 1.042)	0.907 (0.536, 1.535)
South Africa	1.067 (0.952, 1.196)	0.977 (0.882, 1.082)	0.917 (0.684, 1.229)

Table S4. Estimated risk ratios (and 95% confidence intervals) of childhood anemia per 1°C increase in annual temperature in sub-Saharan Africa.

Country	Total anemia	Mild anemia	Moderate anemia	Severe anemia
Eastern Africa				
Burundi	1.029 (1.018, 1.040)	1.073 (1.036, 1.110)	1.046 (1.032, 1.06)	1.010 (0.997, 1.024)
Ethiopia	1.015 (1.010, 1.019)	1.019 (1.007, 1.031)	1.02 (1.015, 1.025)	1.009 (1.004, 1.014)
Malawi	1.049 (1.040, 1.059)	1.042 (1.011, 1.073)	1.055 (1.044, 1.066)	1.043 (1.031, 1.054)
Mozambique	1.036 (1.019, 1.052)	1.036 (0.988, 1.082)	1.035 (1.016, 1.053)	1.036 (1.016, 1.056)
Rwanda	1.052 (1.034, 1.069)	1.070 (0.963, 1.178)	1.090 (1.064, 1.116)	1.027 (1.007, 1.047)
Tanzania	1.044 (1.038, 1.050)	1.050 (1.025, 1.075)	1.054 (1.047, 1.061)	1.036 (1.029, 1.043)
Uganda	1.056 (1.043, 1.070)	1.069 (1.030, 1.108)	1.073 (1.057, 1.089)	1.030 (1.014, 1.046)
Zambia	1.021 (1.001, 1.040)	1.114 (1.042, 1.184)	1.038 (1.015, 1.061)	0.998 (0.975, 1.021)
Zimbabwe	0.999 (0.987, 1.010)	1.014 (0.955, 1.073)	1.006 (0.992, 1.021)	0.996 (0.982, 1.010)
Central Africa				
Angola	1.031 (1.022, 1.041)	1.017 (0.986, 1.048)	1.032 (1.021, 1.043)	1.029 (1.018, 1.040)
Cameroon	1.027 (1.021, 1.033)	0.994 (0.973, 1.015)	1.003 (1.023, 1.037)	1.023 (1.016, 1.029)
Congo Democratic Republic	1.045 (1.041, 1.049)	1.055 (1.042, 1.068)	1.051 (1.046, 1.056)	1.033 (1.028, 1.038)
Gabon	1.074 (1.031, 1.116)	1.089 (0.919, 1.234)	1.057 (1.006, 1.106)	1.091 (1.039, 1.140)

Western Africa				
Benin	1.031 (1.018, 1.044)	1.091 (1.061, 1.119)	1.028 (1.013, 1.043)	1.025 (1.008, 1.041)
Burkina Faso	1.007 (0.994, 1.019)	1.031 (1.013, 1.046)	1.007 (0.993, 1.019)	1.003 (0.987, 1.018)
Cote d'Ivoire	1.005 (0.969, 1.039)	1.010 (0.918, 1.087)	1.012 (0.973, 1.049)	0.994 (0.950, 1.034)
Gambia	1.091 (1.063, 1.119)	1.194 (1.067, 1.311)	1.141 (1.106, 1.176)	1.047 (1.013, 1.081)
Ghana	1.002 (0.971, 1.032)	0.978 (0.901, 1.047)	1.008 (0.973, 1.042)	0.994 (0.957, 1.030)
Liberia	1.067 (0.99, 1.133)	1.246 (1.074, 1.327)	1.064 (0.976, 1.137)	1.053 (0.956, 1.134)
Nigeria	1.030 (1.020, 1.040)	1.024 (0.998, 1.049)	1.031 (1.020, 1.042)	1.029 (1.018, 1.041)
Senegal	1.003 (0.998, 1.008)	1.030 (1.016, 1.044)	1.006 (1.000, 1.011)	0.998 (0.992, 1.004)
Togo	0.980 (0.949, 1.011)	0.916 (0.810, 1.012)	0.968 (0.932, 1.003)	1.001 (0.964, 1.035)
Southern Africa				
Eswatini	1.022 (0.995, 1.049)	0.985 (0.845, 1.129)	1.028 (0.995, 1.060)	1.016 (0.986, 1.046)
Lesotho	0.959 (0.934, 0.985)	0.905 (0.799, 1.014)	0.958 (0.926, 0.990)	0.964 (0.932, 0.995)
Namibia	0.954 (0.915, 0.992)	0.924 (0.665, 1.200)	0.968 (0.920, 1.016)	0.946 (0.901, 0.991)
South Africa	1.010 (0.956, 1.065)	0.943 (0.774, 1.126)	0.985 (0.923, 1.047)	1.042 (0.973, 1.113)

Table S5. Odds ratios (95% confidence intervals) of childhood anemia per 1°C increment in annual temperature for different exposure windows in sub-Saharan Africa.

Model	Total	Mild anemia	Moderate anemia	Severe anemia
Main model	1.138 (1.134, 1.142)	1.090 (1.085, 1.095)	1.173 (1.169, 1.178)	1.259 (1.245, 1.273)
Lag 01	1.093 (1.090, 1.096)	1.067 (1.063, 1.071)	1.110 (1.106, 1.114)	1.126 (1.116, 1.137)
Lag 03	1.107 (1.104, 1.111)	1.074 (1.07, 1.079)	1.129 (1.125, 1.133)	1.158 (1.147, 1.170)
Lag 06	1.126 (1.123, 1.130)	1.082 (1.078, 1.086)	1.156 (1.152, 1.161)	1.224 (1.212, 1.237)
Lag 09	1.133 (1.128, 1.138)	1.094 (1.085, 1.093)	1.165 (1.158, 1.172)	1.236 (1.246, 1.255)

*Note: *Main model* denotes the average temperature during the past 12 from the interview day in the model. Lag 01, Lag 03, Lag 06, Lag 09, Lag 012 denotes the average temperature during the past 1 month, 3 months, 6 months, 9 months from the interview day in the model.

Table S6. R^2 of models for different exposure windows in sub-Saharan Africa in sensitivity analyses.

Model	Total	Mild anemia	Moderate anemia	Severe anemia
Main model	0.074	0.181	0.108	0.033
Lag 01	0.068	0.158	0.097	0.031
Lag 03	0.068	0.165	0.099	0.031
Lag 06	0.069	0.187	0.105	0.030
Lag 09	0.069	0.168	0.102	0.032

*Note: *Main model* denotes the average temperature during the past 12 from the interview day in the model. Lag 01, Lag 03, Lag 06, Lag 09, Lag 012 denotes the average temperature during the past 1 month, 3 months, 6 months, 9 months from the interview day in the model.

Table S7. Mediation of association between annual temperature and anemia risk in children by malnutrition and malaria.

	Malnutrition	Malaria
Total effect (95% CI)	1.138 (1.134, 1.142)	1.138 (1.134, 1.142)
Direct effect (95% CI)	1.123 (1.118, 1.127)	1.125 (1.118, 1.132)
Indirect effect (Empirical 95% CI)	1.015 (1.010, 1.020)	1.013 (1.008, 1.018)
Percent mediated	11.40%	9.74%

Note: Empirical 95% CI: 95% confidence interval for indirect effect generated from 1000 bootstrap samples. percent mediated: proportion of association between annual temperature and childhood anemia mediated by malnutrition or malaria, calculated as the difference between the high temperature exposure coefficient from the main model and the high temperature exposure coefficient from the model adjusted by malnutrition or malaria (direct effect; further adjusted for malnutrition or malaria) divided by the high temperature exposure coefficient from the main model.

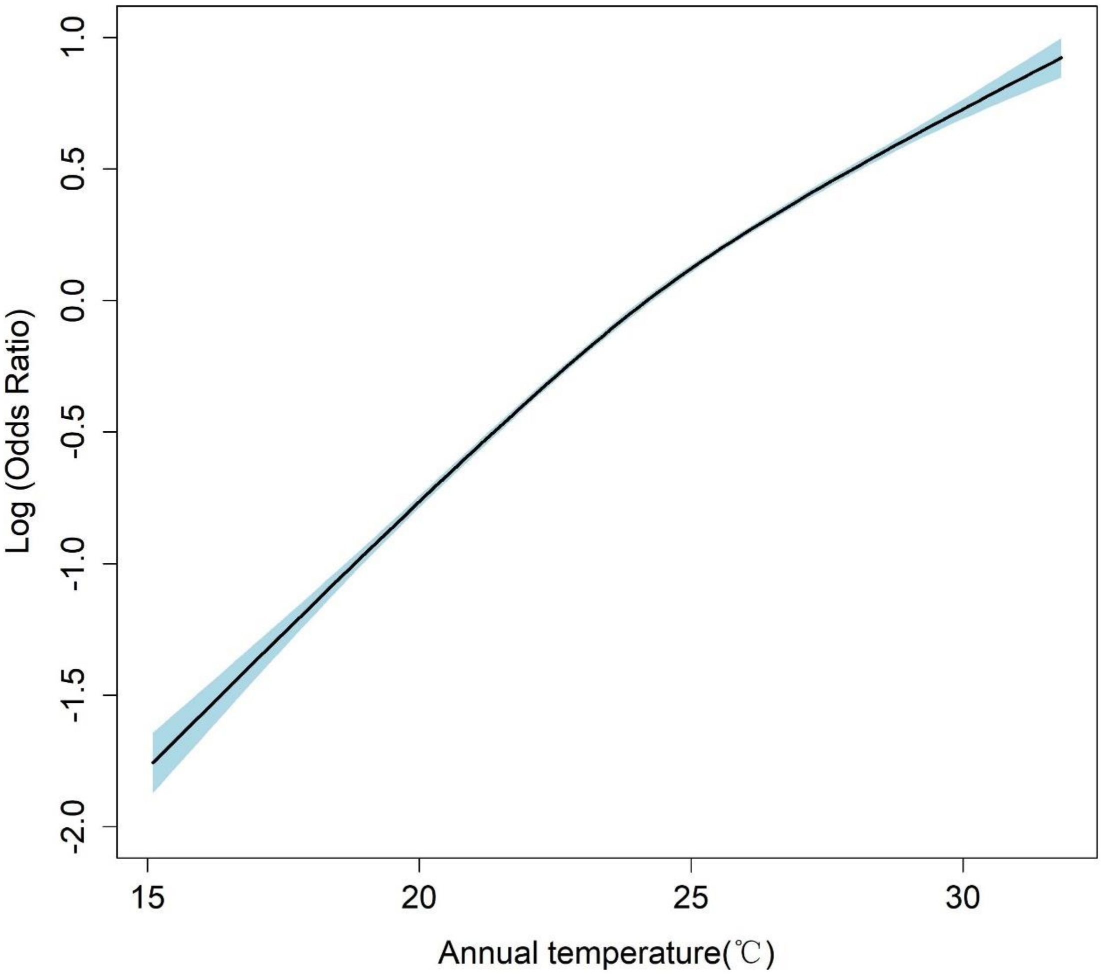

Figure S1. Exposure-response curves for the associations between annual temperature and prevalence of childhood anemia.

The solid line is the log-transformed odds ratio of childhood anemia; the shaded areas is the 95% confidence intervals.

Table S8. Odds ratios (95% confidence intervals) of childhood anemia per 1°C increment in annual temperature in sub-Saharan Africa in four different models.

Model	Total	Mild anemia	Moderate anemia	Severe anemia
Main model	1.138 (1.134, 1.142)	1.090 (1.085, 1.095)	1.173 (1.169, 1.178)	1.259 (1.245, 1.273)
Model 1	1.144 (1.140, 1.147)	1.094 (1.090, 1.098)	1.176 (1.172, 1.180)	1.256 (1.245, 1.268)
Model 2	1.138 (1.134, 1.142)	1.089 (1.085, 1.094)	1.173 (1.168, 1.178)	1.259 (1.245, 1.273)
Model 3	1.138 (1.134, 1.142)	1.090 (1.085, 1.094)	1.173 (1.162, 1.171)	1.260 (1.246, 1.274)
Model 4	1.133 (1.129, 1.137)	1.087 (1.082, 1.091)	1.167 (1.129, 1.137)	1.238 (1.225, 1.252)

[*]Note: the covariates of *Main model* included children's individual factors, socioeconomic factors, country-level covariates, annual cumulative precipitation, survey month and year. The covariates of *Model 1* included the covariates in the *Main model* with the exception of the household factors (wealth level, floor material, roof material, water infrastructures and sanitation infrastructures). The covariates of *Model 2* included the covariates in the *Main model* with the exception of the country-level covariates. The exposure windows for the child less than 365 days spanned from their birth date to the interview date in *Model 4*, while in *Main model*, we calculated annual average temperature for all children under 5 years.

Table S9. Regional odds ratios (95% confidence intervals) of childhood anemia per 1°C increment in annual temperature in sub-Saharan Africa in random effect meta-analysis.

Region	Total anemia	Mild anemia	Moderate anemia	Severe anemia
Eastern Africa	1.085 (1.049, 1.122)	1.054 (1.021, 1.088)	1.116 (1.074, 1.160)	1.124 (1.073, 1.176)
Central Africa	1.121 (1.068, 1.177)	1.096 (1.060, 1.133)	1.125 (1.068, 1.187)	1.087 (0.969, 1.220)
Western Africa	1.073 (1.014, 1.135)	1.051 (1.006, 1.098)	1.087 (1.005, 1.175)	1.171 (1.015, 1.350)
Southern Africa	0.972 (0.910, 1.039)	0.977 (0.907, 1.054)	0.973 (0.911, 1.039)	0.888 (0.772, 1.022)

Table S10. Summary statistics of the projected annual temperature by region, period, and climate change scenario.

Region	Variables	Projected temperature (°C)					
		SSP1-2.6		SSP2-4.5		SSP5-8.5	
		Temperature (°C)	Temperature change (°C) [a]	Temperature (°C)	Temperature change (°C) [a]	Temperature (°C)	Temperature change (°C) [a]
Sub-Saharan Africa	2010s	23.5 (23.3, 23.6)	0.2 (0, 0.3)	23.5 (23.3, 23.6)	0.2 (0, 0.3)	23.5 (23.3, 23.6)	0.2 (0, 0.3)
	2030s	24.1 (23.8, 24.3)	0.8 (0.5, 1.0)	24.0 (23.9, 24.2)	0.7 (0.6, 1.0)	24.1 (23.8, 24.3)	0.8 (0.5, 1.0)
	2050s	24.5 (24.2, 24.8)	1.2 (0.9, 1.5)	24.6 (24.3, 24.8)	1.3 (1.0, 1.5)	25.0 (24.7, 25.5)	1.7 (1.4, 2.2)
	2070s	24.4 (24.2, 24.6)	1.1 (0.9, 1.3)	25.2 (24.9, 25.4)	1.9 (1.6, 2.1)	26.1 (25.5, 26.4)	2.8 (2.2, 3.1)
	2090s	24.4 (24.1, 24.8)	1.1 (0.8, 1.5)	25.5 (25.0, 25.8)	2.2 (1.7, 2.5)	27.3 (26.4, 27.9)	4.0 (3.1, 4.6)
East Africa	2010s	21.5 (21.4, 21.7)	0.2 (0, 0.4)	21.5 (21.4, 21.7)	0.2 (0, 0.4)	21.5 (21.4, 21.7)	0.2 (0, 0.4)
	2030s	22.2 (21.8, 22.5)	0.8 (0.4, 1.1)	22.1 (21.9, 22.4)	0.8 (0.6, 1.0)	22.1 (21.8, 22.3)	0.7 (0.4, 1.0)
	2050s	22.6 (22.4, 22.9)	1.2 (1.0, 1.5)	22.7 (22.3, 23.0)	1.3 (0.9, 1.6)	23.2 (22.8, 23.5)	1.8 (1.4, 2.1)
	2070s	22.5 (22.1, 22.7)	1.1 (0.8, 1.3)	23.2 (22.9, 23.5)	1.9 (1.5, 2.2)	24.1 (23.5, 24.3)	2.7 (2.1, 2.9)
	2090s	22.5 (22.2, 22.8)	1.1 (0.8, 1.4)	23.5 (23.2, 23.9)	2.1 (1.8, 2.5)	25.3 (24.4, 26.1)	4.0 (3.0, 4.7)
West Africa	2010s	27.0 (26.8, 27.1)	0.2 (0, 0.3)	27.0 (26.8, 27.1)	0.2 (0, 0.3)	27.0 (26.8, 27.1)	0.2 (0, 0.3)
	2030s	27.6 (27.4, 27.8)	0.7 (0.6, 1.0)	27.5 (27.2, 27.7)	0.7 (0.4, 0.9)	27.5 (27.3, 27.9)	0.7 (0.5, 1.0)
	2050s	27.9 (27.6, 28.2)	1.1 (0.7, 1.4)	28.0 (27.8, 28.2)	1.1 (0.9, 1.4)	28.4 (28.0, 28.9)	1.6 (1.2, 2.1)
	2070s	27.9 (27.6, 28.4)	1.1 (0.8, 1.5)	28.7 (28.4, 28.9)	1.9 (1.6, 2.1)	29.5 (28.8, 29.8)	2.7 (2.0, 3.0)
	2090s	27.9 (27.6, 28.3)	1.1 (0.8, 1.5)	28.9 (28.5, 29.4)	2.1 (1.7, 2.6)	30.7 (30.0, 31.3)	3.9 (3.2, 4.4)
Central Africa	2010s	23.6 (23.4, 23.7)	0.1 (0, 0.3)	23.6 (23.4, 23.7)	0.1 (0, 0.3)	23.6 (23.4, 23.7)	0.1 (0, 0.3)

	2030s	24.2 (23.8, 24.4)	0.7 (0.4, 1.0)	24.1 (23.9, 24.4)	0.7 (0.5, 1.0)	24.1 (23.7, 24.4)	0.7 (0.3, 1.0)
	2050s	24.5 (24.2, 24.8)	1.0 (0.8, 1.3)	24.6 (24.4, 24.7)	1.2 (1.0, 1.3)	25.0 (24.6, 25.4)	1.6 (1.2, 2.0)
	2070s	24.5 (24.1, 24.6)	1.1 (0.7, 1.2)	25.2 (24.9, 25.4)	1.8 (1.4, 2.0)	25.9 (25.3, 26.3)	2.5 (1.9, 2.9)
	2090s	24.5 (24.1, 24.9)	1.1 (0.7, 1.5)	25.5 (25.0, 25.8)	2.0 (1.6, 2.4)	27.1 (26.2, 27.8)	3.7 (2.7, 4.4)
South Africa	2010s	17.5 (17.2, 17.7)	0.1 (-0.2, 0.4)	17.5 (17.2, 17.7)	0.1 (-0.2, 0.4)	17.5 (17.2, 17.7)	0.1 (-0.2, 0.4)
	2030s	18.2 (17.9, 18.6)	0.8 (0.5, 1.3)	18.2 (17.9, 18.6)	0.9 (0.6, 1.2)	18.2 (17.7, 18.7)	0.9 (0.4, 1.3)
	2050s	18.7 (18.3, 19.2)	1.3 (0.9, 1.9)	18.9 (18.5, 19.3)	1.5 (1.2, 1.9)	19.4 (19.1, 19.7)	2.0 (1.8, 2.3)
	2070s	18.6 (18.1, 18.9)	1.2 (0.8, 1.6)	19.3 (18.9, 19.6)	1.9 (1.5, 2.3)	20.5 (20.1, 20.7)	3.2 (2.7, 3.4)
	2090s	18.5 (18.2, 18.8)	1.2 (0.8, 1.4)	19.7 (19.3, 20.2)	2.4 (1.9, 2.8)	21.9 (21.2, 22.5)	4.6 (3.9, 5.2)

Note: Abbreviation: SSP=Shared Socioeconomic Pathway.

[a] Temperature change means projected temperature increase in the mean temperature versus baseline period (1985-2014).

Table S11. The summary of the projected excess cases of childhood anemia per 100,000 person-year compared to the baseline period (1985–2014) in sub-Saharan Africa by region, period, and climate change scenario, under the assumption of no change in population size and structure.

Region	Variables	Excess cases per 100,000 person-year		
		SSP1-2.6	SSP2-4.5	SSP5-8.5
Sub-Saharan Africa	2010s	425 (109, 730)	425 (109, 730)	425 (109, 730)
	2030s	1,614 (904, 2,293)	1,514 (821, 2,174)	1,450 (755, 2,115)
	2050s	2,312 (1,280, 3,295)	2,440 (1,340, 3,487)	3,268 (1,772, 4,691)
	2070s	2,275 (1,268, 3,236)	3,770 (2,103, 5,358)	5,163 (2,786, 7,425)
	2090s	2,275 (1,277, 3,226)	4,208 (2,311, 6,015)	7,597 (4,133, 10,894)
Eastern Africa	2010s	470 (307, 775)	470 (307, 775)	470 (307, 775)
	2030s	1,951 (1,409, 2,481)	1,934 (1,389, 2,466)	1,779 (1,246, 2,301)
	2050s	2,923 (2,110, 3,717)	3,024 (2,166, 3,863)	4,122 (2,938, 5,279)
	2070s	2,693 (1,940, 3,427)	4,460 (3,209, 5,682)	6,373 (4,562, 8,143)
	2090s	2,691 (1,947, 3,417)	5,039 (3,615, 6,429)	9,276 (6,637, 11,854)
Central Africa	2010s	503 (379, 624)	503 (379, 624)	503 (379, 624)
	2030s	2,227 (1,918, 2,531)	2,080 (1,783, 2,373)	2,037 (1,719, 2,349)
	2050s	3,153 (2,714, 3,584)	3,468 (2,984, 3,944)	4,641 (3,982, 5,290)
	2070s	3,215 (2,769, 3,653)	5,233 (4,501, 5,952)	7,051 (6,036, 8,049)
	2090s	3,229 (2,782, 3,668)	5,916 (5,087, 6,732)	10,566 (9,062, 12,045)
Western Africa	2010s	391 (-31, 753)	391 (-31, 753)	391 (-31, 753)
	2030s	1,421 (539, 2,266)	1,238 (421, 2,019)	1,239 (412, 2,031)
	2050s	1,944 (730, 3,103)	2,048 (779, 3,261)	2,690 (970, 4,331)
	2070s	2,027 (792, 3,208)	3,416 (1,318, 5,422)	4,506 (1,649, 7,231)
	2090s	2,024 (784, 3,208)	3,733 (1,408, 5,953)	6,646 (2,485, 10,619)

Southern Africa	2010s	142 (-489, 658)	142 (-489, 658)	142 (-489, 658)
	2030s	156 (-1,137, 1,322)	183 (-1,203, 1,428)	143 (-1,238, 1,398)
	2050s	213 (-1,870, 2,083)	209 (-2,113, 2,296)	250 (-2,871, 3,051)
	2070s	202 (-1,740, 1,952)	256 (-2,718, 2,928)	322 (-4,434, 4,592)
	2090s	197 (-1,657, 1,864)	279 (-3,378, 3,564)	441 (-6,451, 6,628)

Note: Abbreviation: SSP=Shared Socioeconomic Pathway.

Table S12. The summary of the projected excess cases of childhood anemia per 100,000 person-year compared to the baseline period (1985–2014) in sub-Saharan Africa by region, period, and climate change scenario, under the assumption of no change in population size and structure.

Region	Variables	Excess cases per 100,000 person-year		
		SSP1-2.6	SSP2-4.5	SSP5-8.5
Sub-Saharan Africa	2010s	438 (115, 755)	438 (115, 755)	438 (115, 755)
	2030s	1,734 (1,034, 2403)	1,610 (940, 2,251)	1,545 (866, 2,195)
	2050s	2,516 (1,531, 3,458)	2,680 (1,640, 3,676)	3,574 (2,140, 4,943)
	2070s	2,529 (1,567, 3,452)	4,231 (2,644, 5,753)	5,731 (3,474, 7,889)
	2090s	2,558 (1,616, 3,462)	4,776 (3,005, 6476)	8,578 (5,310, 11,705)
Eastern Africa	2010s	501 (286, 713)	501 (286, 713)	501 (286, 713)
	2030s	1,838 (1,344, 2,321)	1,810 (1,315, 2,294)	1,667 (1,184, 2,139)
	2050s	2,800 (2,057, 3,526)	2,942 (2,154, 3,712)	3,984 (2,901, 5,042)
	2070s	2,630 (1,933, 3,312)	4,444 (3,277, 5,584)	6,199 (4,543, 7,816)
	2090s	2,663 (1,966, 3,343)	5,083 (3,754, 6,381)	9,174 (6,732, 11,557)
Western Africa	2010s	508 (385, 629)	508 (385, 629)	508 (385, 629)
	2030s	2,246 (1,942, 2,545)	2,084 (1,792, 2,371)	2,024 (1,716, 2,327)
	2050s	3,192 (2,760, 3,617)	3,526 (3,046, 3,998)	4,673 (4,025, 5,312)
	2070s	3,245 (2,810, 3,673)	5,286 (4,569, 5,993)	7,088 (6,099, 8,062)
	2090s	3,276 (2,841, 3,704)	6,043 (5,226, 6,847)	10,739 (9,269, 12,187)
Central Africa	2010s	397 (-24, 809)	397 (-24, 809)	397 (-24, 809)
	2030s	1,617 (728, 2,469)	1,384 (567, 2,166)	1,404 (580, 2,196)
	2050s	2,247 (1,082, 3,364)	2,354 (1,124, 3,534)	3153 (1502, 4,733)
	2070s	2,391 (1,213, 3,522)	3,992 (1,977, 5,924)	5,377 (2,678, 7961)
	2090s	2,403 (1,246, 3,514)	4,397 (2,204, 6,501)	8,040 (4,149, 11,773)
Southern Africa	2010s	137 (-751, 1,002)	137 (-751, 1,002)	137 (-751, 1,002)

2030s	260 (-1,713, 2,037)	283 (-1,835, 2,186)	243 (-1,860, 2,154)
2050s	377 (-2,793, 3,223)	340 (-3,213, 3,532)	478 (-4,289, 4,756)
2070s	378 (-2,591, 3,056)	431 (-4,113, 4,510)	659 (-6,575, 7,152)
2090s	404 (-2,429, 2,950)	493 (-5,104, 5,518)	938 (-9,554, 10,356)

Note: Abbreviation: SSP=Shared Socioeconomic Pathway.

Table S13. Proportion of each component and weights of each household asset in each component.

	Component 1	Component 2	Component 3	Component 4	Component 5	Component 6
Proportion of Variance	0.309	0.167	0.119	0.111	0.105	0.085
Cumulative Proportion	0.309	0.476	0.595	0.705	0.81	0.895
Weights						
Electricity	0.329	-0.100	-0.175	0.277	0.037	0.173
Car	0.169	0.329	0.112	0.100	-0.856	-0.438
Television	0.342	-0.037	-0.133	0.251	0.039	0.120
Refrigerator	0.298	-0.088	0.158	-0.061	0.103	0.438
Bicycle	-0.007	0.589	0.042	-0.348	-0.028	0.747
Scooter	0.093	0.495	-0.302	0.123	0.598	-0.558
Mobile telephone	0.172	-0.001	0.804	-0.341	0.281	-0.356
Cooking fuel	-0.153	0.168	0.478	0.851	0.099	0.235

Table S14. Basic information for the twenty general circulation models included in the three scenarios.

Model	Member	Reference
AWI-CM-1-1-MR	r1i1p1f1	[1]
CAMS-CSM1-0	r2i1p1f1	[2]
CanESM5	r1i1p1f1	[3]
CESM2-WACCM	r1i1p1f1	[4]
CMCC-CM2-SR5	r1i1p1f1	[5]
EC-Earth3	r1i1p1f1	[6]
EC-Earth3-Veg	r1i1p1f1	[7]
FGOALS-g3	r1i1p1f1	[8]
GFDL-ESM4	r1i1p1f1	[9]
IITM-ESM	r1i1p1f1	[10]
INM-CM4-8	r1i1p1f1	[11]
KACE-1-0-G	r1i1p1f1	[12]
KIOST-ESM	r1i1p1f1	[13]
MIROC6	r1i1p1f1	[14]
MPI-ESM1-2-HR	r1i1p1f1	[15]
MPI-ESM1-2-LR	r1i1p1f1	[16]
MRI-ESM2-0	r1i1p1f1	[17]
NESM3	r1i1p1f1	[18]
NorESM2-MM	r1i1p1f1	[19]
UKESM1-0-LL	r1i1p1f2	[20]

Supplemental References

1. Semmler, T., Danilov, S., Rackow, T., Sidorenko, D., Barbi, D., Hegewald, J., Pradhan, H.K., Sein, D., Wang, Q., and Jung, T. (2019). AWI AWI-CM1.1MR model output prepared for CMIP6 ScenarioMIP. Earth System Grid Federation.
2. Rong, X. (2019). CAMS CAMS-CSM1.0 model output prepared for CMIP6 ScenarioMIP. Earth System Grid Federation.
3. Swart, N.C., Cole, J.N.S., Kharin, V.V., Lazare, M., Scinocca, J.F., Gillett, N.P., Anstey, J., Arora, V., Christian, J.R., Jiao, Y., et al. (2019). CCCma CanESM5 model output prepared for CMIP6 ScenarioMIP. Earth System Grid Federation.
4. Danabasoglu, G. (2019). NCAR CESM2-WACCM model output prepared for CMIP6 ScenarioMIP. Earth System Grid Federation.
5. Lovato, T., and Peano, D. (2020). CMCC CMCC-CM2-SR5 model output prepared for CMIP6 ScenarioMIP. Earth System Grid Federation.
6. Consortium, E.C.-E. (2019). EC-Earth-Consortium EC-Earth3 model output prepared for CMIP6 ScenarioMIP. Earth System Grid Federation.
7. Consortium, E.C.-E. (2019). EC-Earth-Consortium EC-Earth3-Veg model output prepared for CMIP6 ScenarioMIP. Earth System Grid Federation.
8. Li, L. (2019). CAS FGOALS-g3 model output prepared for CMIP6 ScenarioMIP. Earth System Grid Federation.
9. John, J.G., Blanton, C., McHugh, C., Radhakrishnan, A., Rand, K., Vahlenkamp, H., Wilson, C., Zadeh, N.T., Dunne, J.P., Dussin, R., et al. (2018). NOAA-GFDL GFDL-ESM4 model output prepared for CMIP6 ScenarioMIP. Earth System Grid Federation.
10. Panickal, S., Narayanasetti, S., Gopinathan, P.A., Choudhury, A.D., Singh, M., and Raghavan, K. (2020). CCCR-IITM IITM-ESM model output prepared for CMIP6 ScenarioMIP. Earth System Grid Federation.
11. Volodin, E., Mortikov, E., Gritsun, A., Lykossov, V., Galin, V., Diansky, N., Gusev, A., Kostrykin, S., Iakovlev, N., Shestakova, A., and Emelina, S. (2019). INM INM-CM4-8 model output prepared for CMIP6 ScenarioMIP. Earth System Grid Federation.
12. Byun, Y.-H., Lim, Y.-J., Shim, S., Sung, H.M., Sun, M., Kim, J., Kim, B.-H., Lee, J.-H., and Moon, H. (2019). NIMS-KMA KACE1.0-G model output prepared for CMIP6 ScenarioMIP. Earth System Grid Federation.

13. Kim, Y., Noh, Y., Kim, D., Lee, M.-I., Lee, H.J., Kim, S.Y., and Kim, D. (2019). KIOST KIOST-ESM model output prepared for CMIP6 ScenarioMIP. Earth System Grid Federation.
14. Shiogama, H., Abe, M., and Tatebe, H. (2019). MIROC MIROC6 model output prepared for CMIP6 ScenarioMIP. Earth System Grid Federation.
15. Schupfner, M., Wieners, K.-H., Wachsmann, F., Steger, C., Bittner, M., Jungclaus, J., Früh, B., Pankatz, K., Giorgetta, M., Reick, C., et al. (2019). DKRZ MPI-ESM1.2-HR model output prepared for CMIP6 ScenarioMIP. Earth System Grid Federation.
16. Wieners, K.-H., Giorgetta, M., Jungclaus, J., Reick, C., Esch, M., Bittner, M., Gayler, V., Haak, H., de Vrese, P., Raddatz, T., et al. (2019). MPI-M MPIESM1.2-LR model output prepared for CMIP6 ScenarioMIP. Earth System Grid Federation.
17. Yukimoto, S., Koshiro, T., Kawai, H., Oshima, N., Yoshida, K., Urakawa, S., Tsujino, H., Deushi, M., Tanaka, T., Hosaka, M., et al. (2019). MRI MRI-ESM2.0 model output prepared for CMIP6 ScenarioMIP. Earth System Grid Federation.
18. Cao, J. (2019). NUIST NESMv3 model output prepared for CMIP6 ScenarioMIP. Earth System Grid Federation.
19. Bentsen, M., Oliviè, D.J.L., Seland, Ø., Toniazzo, T., Gjermundsen, A., Graff, L.S., Debernard, J.B., Gupta, A.K., He, Y., Kirkevåg, A., et al. (2019). NCC NorESM2-MM model output prepared for CMIP6 ScenarioMIP. Earth System Grid Federation.
20. Good, P., Sellar, A., Tang, Y., Rumbold, S., Ellis, R., Kelley, D., Kuhlbrodt, T., and Walton, J. (2019). MOHC UKESM1.0-LL model output prepared for CMIP6 ScenarioMIP. Earth System Grid Federation.
